# Sleep Quality and Medication Adherence in Older Adults: A Systematic Review

**DOI:** 10.3390/clockssleep6030032

**Published:** 2024-09-03

**Authors:** Leandro Amato, Noemi Giannetta, Sofia Taborri, Sara Dionisi, Nicolò Panattoni, Emanuele Di Simone, Aurora De Leo, Gloria Liquori, Giovanni Battista Orsi, Fabio Fabbian, Marco Di Muzio

**Affiliations:** 1 Department of Clinical and Molecular Medicine, Sapienza University of Rome, 00185 Rome, Italysofia.taborri@uniroma1.it (S.T.); 2 Departmental Faculty of Medicine, Saint Camillus International University of Health and Medical Sciences (UniCamillus), 00131 Rome, Italy; noemi.giannetta@unicamillus.org; 3 Nursing, Technical, Rehabilitation Department, DaTeR Local Health Unit of Bologna, 40124 Bologna, Italy; sara.dionisi@uniroma1.it; 4 Department of Public Health and Infectious Diseases, Sapienza University of Rome, 00185 Rome, Italy; nicolo.panattoni@uniroma1.it (N.P.); emanuele.disimone@uniroma1.it (E.D.S.); giovanni.orsi@uniroma1.it (G.B.O.); 5 Department of Biomedicine and Prevention, University of Rome Tor Vergata, 00133 Rome, Italygloria.liquori@uniroma1.it (G.L.); 6 Department of Medical Sciences, University of Ferrara, 44121 Ferrara, Italy; f.fabbian@ospfe.it

**Keywords:** sleep quality, medication adherence, elderly, non-adherence

## Abstract

Sleep quality significantly impacts individuals’ overall health, especially in older adults. Older adults often face comorbid conditions, polypharmacy (the intake of five or more medications per day), and medication non-adherence, which are common among those with sleep disorders. The purpose of this systematic review is to examine the relationship between sleep quality and medication adherence in older adults and to describe the measuring instruments used. A systematic review was performed following the PRISMA guidelines. The PubMed, Scopus, Cochrane Library, and CINAHL databases were screened from January 2024 to March 2024. Nine articles were included in the final synthesis based on the inclusion and exclusion criteria. The review found that poor sleep quality is significantly associated with reduced medication adherence in older adults. The key sleep determinants linked to medication non-adherence include sleep disorders, sleep efficiency, sleep duration, and daytime dysfunctions. Inappropriate medication prescriptions have been associated with reduced sleep efficiency. The tools for assessing sleep quality and medication adherence are predominantly subjective and varied. To address these challenges, a comprehensive geriatric assessment should include investigations into sleep disorders and comorbidity factors. Additionally, nursing educational interventions could be pivotal in improving medication adherence among older adults.

## 1. Introduction

Sleep is a crucial indicator of overall health and well-being, representing a fundamental aspect of quality of life, especially in older adults [1]. Sleep quality affects the daily activities of every individual, influencing physical, cognitive, and psychological spheres [2]. The consequences of poor sleep quality are evident in improper daily eating habits; feelings of irritability, perceived exhaustion, or fatigue; and altered physiological responses [3].

Sleep disorders constitute a significant global public health concern [4], primarily characterised by issues related to sleep duration, efficiency, fragmentation, and daytime sleepiness [2]. Ageing brings about physiological changes in the structure and quality of sleep [5,6]. Compromised sleep quality can lead to drowsiness, fatigue, depression, irritability, pain, muscle tremors, and decreased mental and functional abilities in older adults [7]. Frailty, often associated with comorbidities, increases the vulnerability of older adults to physical and psychological stressors related to sleep, worsening their overall health status [8]. The literature indicates that the increase in chronic conditions and the number of medications in older adults are associated with sleep disorders [9]. For these reasons, a bidirectional relationship between sleep disorders and chronic conditions is often highlighted, as sleep is both a risk factor and a consequence of these disorders [2,10,11].

Beyond the presence of comorbidities, older patients are more likely to develop polypharmacy and have a higher risk of medication non-adherence compared to younger individuals [12,13]. The term “polypharmacy” refers to the intake of five or more medications per day in managing a patient’s comorbidities, and it often refers to the consumption or prescription of more drugs than are clinically appropriate in the course of treatment [14].

Medication adherence, defined as a deliberate and proactive decision of a patient to comply with a healthcare provider’s instructions, is an individual behaviour determined by various components, including personal attitude, pharmacological treatment, the healthcare system, and the type of healthcare provided [15]. Medication non-adherence, intentional or unintentional, can manifest as missed or incorrect medication intake and temporary or permanent treatment interruption [15,16]. Older adults are at a high risk of medication non-adherence due to the presence of multiple pathologies [17]; cognitive impairments [18,19]; memory deficits, forgetfulness, or inattention related to physiological ageing [2,15]; and the high number of medications to be taken [13,15].

Poor sleep quality, influencing decision-making processes and complex abilities such as problem-solving, poses a potential risk to individual capacities for health management and self-care [20]. Furthermore, poor sleep quality combined with inadequate self-care increases the number of emergency department visits and the risk of mortality [21]. Reviews focusing on sleep disorders and health management capabilities are lacking and outdated [20]. Thus, this study examined the relationship between sleep quality and medication adherence in older adults. Furthermore, this systematic review aimed to describe the tools used to measure sleep quality and medication adherence and summarise all the sleep quality factors that influence medication adherence.

Based on that, this systematic review sought to answer the following research questions: What is the relationship between sleep quality and medication adherence in older adults? What tools are used to measure sleep quality and medication adherence in the included studies?

## 2. Results

The literature search produced 1156 papers, with 1144 after de-duplication. Based on the titles and abstracts, 1093 articles were excluded. A total of 51 articles were assessed as full text. Of these, 8 were excluded for including a specific population, 15 for not being original research, and 19 for not including sleep measurement tools. Ultimately, nine studies met the criteria for eligibility and were included in the review.

**Figure 1:**
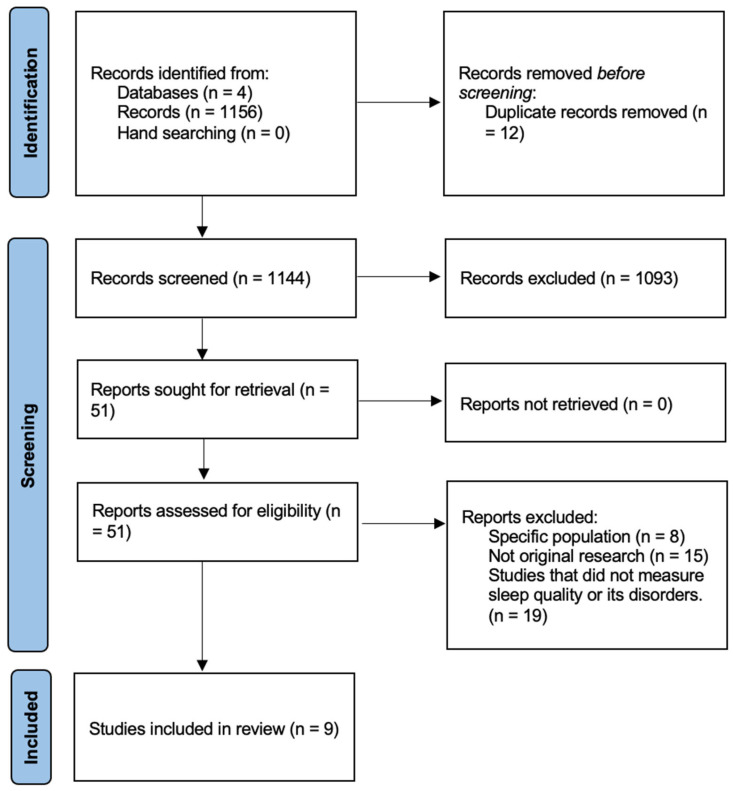
PRISMA flowchart of study selection process.

Figure 1 shows the study’s PRISMA flow chart.

Nine studies [22,23,24,25,26,27,28,29,30] addressed the relationship between sleep quality and medication adherence. Seven cross-sectional studies [22,23,25,26,27,28,29] and two longitudinal studies [24,30] were included. Among these studies, three were conducted in North America (USA) [23,26,30], two in Europe (Switzerland and Poland) [25,29], two in Asia (Malaysia and Japan) [24,28], one in Oceania (Australia) [27], and one in South America (Brazil) [22].

The sample sizes of the studies range from 24 to 2712 participants, with an average age of over 60 years.

Table 1 shows the main characteristics of the studies.

### 2.1. Measurement Tools for Assessing Sleep Quality and Medication Adherence

Regarding sleep measurements, six studies used the Pittsburgh Sleep Quality Index (PSQI) [23,24,26,28,29,30], three studies used the Epworth Sleepiness Scale (ESS) [22,24,26], and one used a four-item Likert scale [25]. The associated subjective scales included the four-item Patient-Reported Outcomes Measurement Information System (PROMIS-SD) [30], the Stanford Sleepiness Scale (SSS) [26], the Insomnia Severity Index [24], the Beck Depression Inventory [27], and the Clinical Interview Scheduled-Revised (CIS-R) [22]. Only two studies used objective measurements through actigraphy to assess sleep quality in association with subjective measurements [22,24].

To assess medication adherence, the tools used included the Morisky Medication Adherence Scale (MMAS-4) [22], the Adherence to Refills and Medication Scale (ARMS) [25], the Medication Event Monitoring System (MEMS) [26], the Drug Burden Index (DBI) [28], the Medication Adherence Report Scale (MARS-5) [27], the Ask-12 Medication Survey [30], medication intake interviews [29], and physiological parameters [24].

Table 2 shows descriptions of the measurement tools included in the studies.

### 2.2. Factors of Sleep Quality That Influence Medication Adherence

In all of the included studies, the sample with poor sleep quality was more likely to report reduced medication adherence compared to the good sleepers group [22,23,24,25,26,27,28,29,30]. Le Grande et al. showed that the association between sleep disorders and medication adherence became significant after four months of observation [27], while Telford et al. demonstrated that the likelihood of non-adherence increased by 9% for each point increase on the PSQI scale [23].

Among the determinants of sleep quality associated with medication non-adherence were sleep disorders [23], sleep efficiency [28,29], sleep duration [26], and daytime dysfunctions [22,23,28]. In Kim’s study, sleep disorders were associated with lower medication adherence (*p* < 0.001) [30]. However, Telford’s study did not find evidence that sleep disorders have a mediated effect on treatment adherence (*p* < 0.25) despite their significant association (*p* < 0.003) [23].

In Simoes Maria’s study, sleep efficiency positively correlates with good self-rated health by patients (*p* = 0.004), which, in turn, is negatively associated with medication use (*p* < 0.001) [29]. In Aielo’s study, excessive daytime sleepiness was associated with a higher Body Mass Index (BMI) and a greater frequency of abdominal obesity in patients [22].

Besides poor sleep quality, older age [26], worse clinical conditions, longer hospitalisation periods, and poorer quality of daily functioning were associated with poor medication adherence [25]. Knafl et al. identified three pairs of interacting risk factors capable of increasing the likelihood of medication non-adherence [26]. These included the presence of multiple pathologies in association with polypharmacy, advanced age with worse overall sleep quality, and a shorter period since diagnosis correlated with poorer sleep quality [26].

Regarding pharmacotherapy, Kumar et al. highlighted that prescribing inappropriate medications is associated with reduced sleep efficiency (*p* = 0.037) [28]. The self-assessment of one’s health status in older patients with comorbidities may be negatively influenced by the adverse effects of some medications on sleep quality [29]. For these reasons, sleep disorders have been described as a probable risk factor for poor self-management capacity (*p* < 0.001) [30]. Additionally, Kumar et al. suggested that older patients with poor sleep quality tend to resort more to sedative medications, which increases the risk of developing sleep-related disorders (such as excessive daytime sleepiness) and experiencing falls or fractures [28].

### 2.3. Quality of Evidence

The GRADE evaluation rated all endpoints as moderate, low, or low-quality evidence. The authors consistently downgraded by one point each for study design limitations (as all studies in this review were non-randomised) and inconsistency (Table 3).

In the risk of bias and certainty assessment, none of the studies achieved a high certainty rating. Four studies were rated as having low certainty, while five were rated as having very low certainty. Trends indicate that many studies have a low level of certainty primarily due to serious risks of bias and imprecision in the data. In particular, studies that show a non-serious risk of bias and no imprecision still often have low certainty. This suggests that variability in the data and the quality of observational studies negatively impact the overall robustness of the conclusions.

## 3. Methods

### 3.1. Study Design

This systematic review was performed according to the Preferred Reporting Items for Systematic Reviews and Meta-Analyses (PRISMA) guidelines [31].

#### 3.1.1. Database and Search Strategy

The articles included in this review were identified through screening the PubMed, Scopus, Cochrane Library, and CINAHL databases. The search was conducted between January 2024 and March 2024. In addition, a hand search was conducted to identify any further relevant studies that might not have been captured through the initial search strategy. This involved reviewing the reference lists of included studies and key reviews identified during the initial screening process. However, no additional relevant studies were identified beyond those already included in this review. A search strategy was drawn up according to population, exposure, and outcome system (PEOS) (Table 4).

The search terms used were as follows: “medication non-adherence”, “poor medication adherence”, “poor medication compliance”, “noncompliance”, “treatment refusal”, “sleep deprivation”, “sleep debt”, “sleep disrupted”, “sleep disturbance”, “sleep disorder”, “sleep quality”, “elderly”, “aged”, “older”, “elder”, “geriatric”, “senior”, and “older adults”.

#### 3.1.2. Eligibility Criteria and Study Selection

All types of study designs written in the English or Italian languages were included. The time frame covered the past ten years (2014–2024). The selected studies provided assessment measures regarding sleep quality and medication adherence. Older populations with comorbidities or chronic conditions were analysed.

Exclusion criteria included the following: paediatric and adolescent populations, studies lacking sleep quality and medication adherence measurements, and non-original research.

#### 3.1.3. Data Extraction and Quality Assessment

For each article included, two authors independently extracted the following information using a standardised data abstraction form: author, year, journal, title, study design, sample, the objective of the study, and main results. The evidence quality was measured using the Grading of Recommendations, Assessment, Development and Evaluations (GRADE) method using the software GRADEproGDT, (© 2021, McMaster University and Evidence Prime Inc., Krakow, Poland) and the quality assessment of the relevant studies is reported in Table 3.

## 4. Discussion

This systematic review aimed to examine the relationship between sleep quality and medication adherence in older adults and to describe the measuring instruments used.

The review identified a consistent association between poor sleep quality and reduced medication adherence across various studies. Specifically, sleep disorders, sleep efficiency, sleep duration, and daytime dysfunctions significantly impacted adherence to pharmacological treatments.

In Kim and Telford’s studies, sleep disorders were associated with lower medication adherence [30] or non-adherence [23] and a reduced ability to self-manage chronic conditions [30].

In Kumar’s study, sleep efficiency decreases in the presence of inappropriate medication treatment [28]. Differently, in Simoes Maria’s study, sleep efficiency positively correlates with good self-rated health by patients, which is negatively associated with medication use [29]. All of this suggests that good sleep efficiency promotes feeling healthy, while using medications in the presence of comorbidities can negatively affect the perception of one’s health and sleep hygiene [29].

Our study’s measurement instruments for sleep quality and medication adherence are heterogeneous. Among subjective assessment scales of sleep quality, the most used was the Pittsburgh Sleep Quality Index (PSQI) [23,24,26,28,29,30], followed by the Epworth Sleepiness Scale (ESS) [22,24,26]. Only in the studies by Sakamoto and Aielo were subjective scales complemented by an objective evaluation tool, such as actigraphy [22,24]. In Le Grande’s study, sleep assessment was conducted through an adaptation of the Beck Depression Inventory, which properly assesses the severity of depressive symptoms [27]; similarly, in Aielo’s study, sleep assessment was integrated using the Clinical Interview Scheduled Revised (CIS-R), which evaluates depressive disorders [22]. The most used tools for assessing medication adherence are scales that investigate patients’ behaviour or the frequency and appropriateness of medication administration. In Sakamoto’s study, adherence is inferred from the measurement of physiological parameters, such as body mass index, systemic blood pressure, triglyceride level, etc. [24], while in Simoes Maria’s study, adherence is assessed through interviews conducted with patients about their medication-taking habits [29].

Sleep disorders are associated with a deterioration in patients’ quality of life [22]. Disturbed sleep negatively affects cognitive and executive functions, tasks requiring prolonged attention and processing capacity, and can reduce the involvement and motivation of the individual to engage in behaviours useful for improving their health status [30].

The interaction of multiple risk factors, such as advanced age, polypharmacy, sleep disorders, multiple health conditions, and the experience of illness, puts individuals at risk of medication non-adherence [26,28]. Pharmacological appropriateness, for example, analysed in the context of multimorbidity and advanced age, becomes challenging to manage, and this can lead to inappropriate prescriptions [28]. The search for modifiable risk factors associated with sleep disorders is a fundamental objective in poor medication adherence [26]. Although several subjective factors, such as attitude, personality, or personal motivation, can influence adherence to pharmacological treatment, sleep can be beneficial in improving treatment understanding and compliance [32], as it supports functions like memory and learning [33].

Compromised sleep can be viewed as part of a multifaceted geriatric syndrome, capable of increasing the risk of mortality [34], cardiovascular events, and traumatic falls [35,36], thereby reducing the quality of life of older adults rather than being seen simply as a product of ageing [11].

For a comprehensive geriatric assessment and proper sleep hygiene, the literature recommends investigating sleep disorders and conditions of comorbidity or other factors that can influence sleep, such as pharmacotherapy [13,37] and environmental and psychosocial factors; conducting physical examinations and targeted investigations into sleep disorders by considering objective tools like polysomnography (PSG); and implementing interventions to optimise underlying comorbidities (e.g., the presence of pain, exacerbating medications, reducing environmental stimuli, etc.) to improve medication adherence [11].

Most studies suggest that treating sleep disorders improves the overall quality of life in older patients [38,39,40]. The literature has shown that increased physical exercise in older patients is correlated with better sleep quality [24], and high sleep efficiency is associated with a good self-assessment of one’s health [29].

The management of therapeutic regimens for older patients also involves healthcare professionals and caregiving activities [41,42]. Nursing educational interventions based on proper sleep hygiene, a balanced diet, a healthy lifestyle, and medication adherence management programmes can improve self-management behaviours regarding chronic diseases in older patients [24,43].

Healthcare providers should prioritise the assessment of sleep quality as part of routine geriatric care. Evaluating sleep quality through objective measurement scales could help increase accuracy in investigating disorders and refining corrective treatments. Interventions to improve sleep, such as cognitive behavioural therapy for insomnia (CBT-I) and medication adjustments, could enhance adherence to treatment regimens. Tailored patient education on sleep hygiene, combined with support for medication management, may also prove beneficial.

### Limitations

Our study has limitations. Firstly, most articles included in this systematic review have a low quality level. One of the key findings is that the majority (seven out of nine) of the selected studies were cross-sectional in design. The predominance of cross-sectional studies has implications for the overall interpretation of our results. The studies reflect specific populations or contexts at a single point in time. This constraint should be considered when applying our findings to broader populations or different settings. In addition, the studies included sampled older adults with various comorbidities and analysed them using different study methods, increasing the variability of results across studies, which may limit the generalizability of the findings. Given the heterogeneity of the tools used to measure therapeutic adherence, the association with sleep quality is not standardised. Moreover, subjective scales primarily investigate sleep quality, which may affect the overall sleep assessment.

## 5. Conclusions

Sleep disturbances represent an important risk factor in the older population, which often takes many medications due to the high number of comorbidities. Given the association between sleep and medication adherence, it is useful to investigate sleep quality and its compromise in a broader geriatric assessment context. Due to the predominance of cross-sectional and low-quality studies included in this review, the conclusions drawn should be approached with caution. Future research should prioritise longitudinal studies to explore temporal dynamics and to increase the generalizability of the results. Promoting education aimed at preserving good sleep quality and exploring the interaction of multiple connected risk factors could have benefits on medication adherence and the quality of life of older patients.

## Figures and Tables

**Table 1:** Characteristics of included studies investigating relationship between sleep quality and medication adherence.

Authors	Year	Journal	Title	Study Design	Sample	Objective of the Study	Main Results
Aielo et al. [22]	2023	*Sleep Medicine*	Excessive daytime sleepiness, but not sleep apnea, sleep duration or insomnia, was associated with poor adherence to anti-hypertensive treatment: The ELSA-Brasil study.	Secondary analysis from a cohort study (cross-sectional).	411 patients with hypertension.	To evaluate the impact of sleep disorders on adherence to antihypertensive therapy.	A medium-low medication adherence to the anti-hypertensive treatment was significantly associated with excessive daytime sleepiness (OR: 1.63; 95% IC: 1.05–2.53).
Telford et al. [23]	2020	*Chronic Illn.*	The relationship between Pittsburgh Sleep Quality Index subscales and diabetes control.	Secondary analysis from an RCT (cross-sectional).	281 patients with type II diabetes.	To explore the relationship between self-reported sleep quality and diabetes control.	Sleep disorders and daytime dysfunctions were associated with medication non-adherence, which was more likely to be found in poor sleepers (OR 2.04, 95% IC: 1.27–3.30).
Sakamoto et al. [24]	2021	*BMJ Open*	Can a sleep disorder intervention-embedded self-management programme contribute to improve management of diabetes? A pilot single-arm pretest and post-test study.	A single-arm pre-test and post-test study (longitudinal).	24 patients with diabetic nephropathy.	To evaluate the effectiveness of a diabetes self-management programme incorporating measures to improve sleep and increase physical activity.	Increased physical exercise was correlated with better sleep quality. Nursing training improved scores related to quality of life and self-management behaviours.
Polański et al. [25]	2020	*Advances in Experimental Medicine and Biology*	Medication compliance in COPD patients.	Retrospective study (cross-sectional).	106 old patients with chronic obstructive pulmonary disease.	To determine the influence of the selected variables on adherence to pharmaceutical treatment in patients with chronic obstructive pulmonary disease.	Worse clinical conditions with poor medication adherence were associated with older age (*p* = 0.020), longer hospitalisation periods (*p* = 0.046), and poorer sleep quality (*p* = 0.008) and daily functioning (*p* = 0.001).
Knafl et al. [26]	2014	*Patient Preference and Adherence*	What puts heart failure patients at risk for poor medication adherence?	Secondary analysis from a prospective cohort study (cross-sectional).	280 patients with heart failure.	Identifying a model of risk factors for poor therapeutic adherence.	The presence of multiple comorbidities in association with polypharmacy; older age with poorer overall sleep quality; and less illness experience related to worse sleep quality increased the risk of medication non-adherence.
Le Grande et al. [27]	2015	*Psychology, Health & Medicine*	Relationship between sleep disturbance, depression and anxiety in the 12 months following a cardiac event.	Secondary analysis from an RCT (cross-sectional).	104 patients with heart disease.	To assess the relationship between sleep disturbances, therapeutic adherence, self-efficacy, anxiety, and depression.	Sleep disorders were associated with poor medication adherence at four months of observation and with high anxiety and depression scores at twelve months of observation.
Kumar et al. [28]	2019	*PloS ONE*	The relationship between sleep quality, inappropriate medication use and frailty among older adults in aged care homes in Malaysia.	Descriptive observational study (cross-sectional).	135 polymedicated elderly patients.	To determine the associations between sleep quality, inappropriate medication use, and frailty in older adults.	Inappropriate medication prescriptions were correlated with lower sleep efficiency (*p* = 0.037), poorer subjective sleep quality (*p* = 0.045), and increased use of sedative medications to facilitate sleep (*p* = 0.001).
Simoes Maria et al. [29]	2019	*European Geriatric Medicine*	Sleep characteristics and self-rated health in older persons.	Descriptive study (cross-sectional).	2712 older patients.	To examine the association between sleep characteristics and self-rated health.	Good sleep efficiency was positively correlated with good self-rated health, while the use of medications was negatively associated with good self-rated health.
Kim et al. [30]	2023	*Research Square*	Trajectories of Sleep Disturbance and Self-Management of Chronic Conditions during COVID-19 among Middle-aged and Older Adults.	Cohort study (longitudinal).	549 patients with chronic conditions.	To evaluate trajectories of sleep disturbance and their associations with one’s capacity to self-manage chronic conditions.	The high probability of developing sleep disorders was associated with lower medication adherence and reduced effectiveness in self-managing chronic conditions (*p* < 0.001).

**Table 2:** Measurement tools used to assess sleep quality and medication adherence.

	Measurement Tools	Description	Studies
Sleep Quality	Pittsburgh Sleep Quality Index (PSQI)	Nineteen items assessing subjective sleep quality over the past month. The determinants include duration, quality, sleep disturbances, latency, use of sleep medications, and the impact of sleep on daily life.	[23,24,26,28,29,30]
	Epworth Sleepiness Scale (ESS)	Eight items on the subjective assessment of daytime sleepiness and propensity to fall asleep during the day.	[22,24,26]
	Four-item Likert scale	Four items that estimate the subjective impact of the chronic condition on sleep (from mild to very significant).	[25]
	Four-item Patient-Reported Outcomes Information System (PROMIS-SD)	Four items for a multidimensional and subjective assessment of sleep quality. Determinants include sleep disturbances and daytime sleepiness.	[30]
	Stanford Sleepiness Scale (SSS)	Seven levels expressing the subjective level of sleepiness at a specific moment (from “completely awake” to “asleep”).	[26]
	Insomnia Severity Index	Seven items to assess the subjective severity of insomnia. Determinants include difficulty falling asleep, sleep maintenance, early morning awakening, and daytime functioning.	[24]
	Beck Depression Inventory	Twenty-one items to assess the severity of depression. Determinants include loss of interest in activities, changes in sleep, fatigue, difficulty concentrating, etc.	[27]
	Clinical Interview Scheduled Revised (CIS-R)	Clinical interview to investigate psychiatric symptoms. Domains include depressive symptoms, sleep disturbances, eating disorders, etc.	[22]
	Actigraphy	A technique used to assess the objective quality of sleep through body movement analysis and nocturnal activity pattern studies.	[22,24]
Medication Adherence	Morisky Medication Adherence Scale (MMAS-4)	Four items assessing patient behaviour regarding medication intake in terms of frequency and consistency of intake.	[22]
	Adherence to Refills and Medication Scale (ARMS)	Assesses patient behaviour during medication therapy, including adherence to medical prescriptions.	[25]
	Medication Monitoring System (MEMS)	An electronic system capable of recording the time and date of opening/closing of the container containing the medication to be taken.	[26]
	Drug Burden Index (DBI)	A tool that estimates the adverse effects of multiple drugs on the individual.	[28]
	Medication Adherence Report Scale (MARS-5)	Five items to assess adherence to pharmacological therapy. Determinants include frequency and appropriateness of administration, therapy discontinuation, and communication with the doctor about the treatment to be taken.	[27]
	Ask-12 Medication Survey	Twelve items to assess adherence to pharmacological therapy. Determinants include frequency and appropriateness of administration, problems in medication intake, and communication with the doctor about the treatment to be taken.	[30]
	Medication intake interview	Interview on medication intake habits to promote sleep.	[29]
	Physiological parameters	Adaptation of pharmacological therapy based on physiological indicators, such as body mass index, systemic blood pressure, triglyceride level, etc.	[24]

**Table 3:** GRADE assessment.

Certainty Assessment							№ of Patients		Certainty
№ of Studies	Study Design	Risk of Bias	Inconsistency	Indirectness	Imprecision	Other Considerations	Intervention	Control	
1 [22]	observational studies	not serious	not serious	not serious	not serious	none	156 (38%) high adherence	255 (62%) medium/low adherence	⨁⨁◯◯ LOW
1 [23]	observational studies	serious ^a^	not serious	not serious	not serious	none	97 (34.5%) non-adherent	182 (64.8%) adherent	⨁◯◯◯ VERY LOW
1 [24]	observational studies	not serious	not serious	not serious	serious ^b^	none	24/26 (92.3%)	-	⨁◯◯◯ VERY LOW
1 [25]	observational studies	not serious	not serious	not serious	not serious	none	91 (85.9%) low adherence	15 (14.1%) high adherence	⨁⨁◯◯ LOW
1 [26]	observational studies	not serious	not serious	not serious	not serious	none	218/242 (90.1%)	-	⨁⨁◯◯ LOW
1 [27]	observational studies	not serious	serious ^c^	not serious	not serious	none	107/134 (79.8%)	-	⨁◯◯◯ VERY LOW
1 [28]	observational studies	serious ^a^	not serious	not serious	not serious	none	135	-	⨁◯◯◯ VERY LOW
1 [29]	observational studies	serious ^a^	not serious	not serious	not serious	none	2700	-	⨁◯◯◯ VERY LOW
1 [30]	observational studies	not serious	not serious	not serious	not serious	none	549	-	⨁⨁◯◯ LOW

Explanations: ^a^: Inadequate control for confounders. ^b^: Total numbers < 100 patients. ^c^: Lack of information on follow-up.

**Table 4:** PEOS.

PEO System	Inclusion Criteria	Exclusion Criteria
P—Population	Older adults	Children, adults < 65 years
E—Exposure	Sleep disorders	No sleep quality evaluation
O—Outcome	Medication adherence	No medication adherence evaluation
